# Dynamic Evolution of Avian RNA Virus Sensors: Repeated Loss of RIG-I and RIPLET

**DOI:** 10.3390/v15010003

**Published:** 2022-12-20

**Authors:** Veronika Krchlíková, Tomáš Hron, Martin Těšický, Tao Li, Lenka Ungrová, Jiří Hejnar, Michal Vinkler, Daniel Elleder

**Affiliations:** 1 Institute of Molecular Genetics of the Czech Academy of Sciences, 14220 Prague, Czech Republic; 2 Department of Zoology, Faculty of Science, Charles University, 12843 Prague, Czech Republic

**Keywords:** avian genome, viral sensors, innate immunity, gene loss

## Abstract

Retinoic acid-inducible gene I (RIG-I) and melanoma differentiation-associated protein 5 (MDA5) are key RNA virus sensors belonging to the RIG-I-like receptor (RLR) family. The activation of the RLR inflammasome leads to the establishment of antiviral state, mainly through interferon-mediated signaling. The evolutionary dynamics of RLRs has been studied mainly in mammals, where rare cases of RLR gene losses were described. By in silico screening of avian genomes, we previously described two independent disruptions of MDA5 in two bird orders. Here, we extend this analysis to approximately 150 avian genomes and report 16 independent evolutionary events of RIG-I inactivation. Interestingly, in almost all cases, these inactivations are coupled with genetic disruptions of RIPLET/RNF135, an ubiquitin ligase RIG-I regulator. Complete absence of any detectable RIG-I sequences is unique to several galliform species, including the domestic chicken (*Gallus gallus*). We further aimed to determine compensatory evolution of MDA5 in RIG-I-deficient species. While we were unable to show any specific global pattern of adaptive evolution in RIG-I-deficient species, in galliforms, the analyses of positive selection and surface charge distribution support the hypothesis of some compensatory evolution in MDA5 after RIG-I loss. This work highlights the dynamic nature of evolution in bird RNA virus sensors.

## 1. Introduction

In vertebrates, pattern recognition receptors (PRR) form the first line of defense against invading pathogens. PRRs recognize pathogen-associated molecular patterns (PAMPs) present on the pathogen or generated during its replication in the host cell. PAMPs represent a diverse array of molecules (e.g., DNA or RNA structures, or bacterial lipopolysaccharide) that are not found in the host under normal circumstances or that are mislocalized [1,2]. When PAMPs are detected, PRRs trigger the pathogen-specific production of interferons and other cytokines, which in turn leads to targeted expression of various effector molecules, including interferon-stimulated genes (ISGs) involved in the immune defense.

Several PRRs are involved in viral RNA recognition in birds [3]. Most importantly, RNA-sensing PRRs include RIG-I-like receptors (RLRs), which are cytosolic RNA sensors responsible for the detection of viral or other atypical RNA [4,5]. The RLRs, a helicase family, consist of three structurally similar proteins: retinoic acid-inducible gene I (RIG-I or DDX58) [6], melanoma differentiation-associated protein 5 (MDA5) [7] and laboratory of genetics and physiology 2 (LGP2) [8]. Each of these three proteins consists of a central helicase domain and a carboxy-terminal domain (CTD); RIG-I and MDA5 also encode two amino-terminal caspase activation and recruitment (CARD) domains. The helicase and CTD domains participate directly in RNA binding while the CARD domains are responsible for downstream signaling. LGP2, which lacks the two CARD domains, has been proposed to fulfill the modulatory function by downregulating the signaling through the other two RLR sensors [9]. Furthermore, RIG-I can become involved in a sensory complex of the RIG-I inflammasome to facilitate sensing of cytosolic viral infections [10]. 

Even though RIG-I and MDA5 both recognize extrinsic RNAs, their sensing function is mostly non-redundant [11,12]. RIG-I specifically recognizes exogenous RNAs by their 5’ end, where host RNAs mostly contain a cap structure or a single phosphate. In contrast, exogenous RNAs may have a diphosphate or a triphosphate at their 5′ terminus, and are usually not methylated at the 2′-O position of their first nucleotide [13,14,15,16]. In comparison to RIG-I, which mostly detects shorter RNAs, MDA5 preferentially recognizes longer double-stranded RNAs (dsRNAs) and internal duplex structures in the RNAs [17,18]. There are many layers of post-translational control of RLR sensors; for RIG-I, one of the main modifications is ubiquitylation by RIPLET/RNF135 [1,12,19].

The constant evolution and continuing arms race between pathogens and host defense mechanisms have led to strong patterns of positive selection detected in both RIG-I and MDA5. The positively selected sites (PSSs) were detected in the functional domains as well as in RNA binding sites of both proteins [20,21]. In general, the pathogen-related selection forces can also lead to gene gain (e.g., by gene duplication, leading to the creation of multiprotein families) or to gene loss [22,23]. Although RIG-I and MDA5 seem to play unique roles in RNA detection, the loss of one of these genes has previously, albeit rarely, been reported in mammals, including tree shrews and pangolins [24,25]. This is similar to the evolution of other key PRRs [26,27,28]. In birds, RLR loss was first described in chicken, where RIG-I absence was linked to the increased sensitivity to the influenza virus infection [29,30]. Avian RLRs were further identified and analyzed using bioinformatic tools in a genomic dataset of 62 species [31]. That work focused mainly on the description of selection pressure, but it also made preliminary claims suggesting multiple losses of RLRs. 

In our previous study, we described MDA5 loss in two avian orders, Ciconiiformes and Gruiformes, based on a computational screening of approximately 100 avian species’ genomes [32]. Here, we extend that dataset and describe multiple evolutionary losses of RIG-I and of its regulatory ubiquitin ligase RIPLET. To infer the putative functional impact of the RIG-I loss, we also focus on possible compensatory selection acting on MDA5.

## 2. Materials and Methods

### 2.1. Identification of Avian MDA5, RIG-I and RIPLET Coding Sequences

*RIG-I* and *MDA5* coding sequences from 149 avian species were collected (File S1). These included our already published dataset from 101 species [32] and sequences from 48 additional species. These sequences were either obtained from the NCBI assembled genome database or identified de novo by screening and assembling of the “raw” sequencing data from the NCBI SRA database. This was performed in a similar way to that already described [32]. In species possessing intact gene orthologs, we were able to identify full-length coding sequences (CDS). The only exceptions were speckled mousebird (*Colius striatus*) and northern fulmar (*Fulmarus glacialis*), where we were unable to assemble a part of the first exon of *MDA5* gene. In addition, intact (File S1) and disrupted *RIPLET* coding sequences were identified, similarly to for RIG-I and MDA5. 

To declare a particular ortholog as nonfunctional, it was necessary for it to contain at least one premature stop codon or frameshift mutation or large deletion affecting a significant portion of the sequence. Although cryptic pseudogenization events [33] cannot be identified using this approach, this is the only standardized procedure for mapping gene loss events in large comparative datasets. In all cases of nonfunctional orthologs, we observed more than one deleterious mutation. The mutations were confirmed using public data from at least two independent sources (e.g., assembled WGS data and short reads from RNA sequencing). 

### 2.2. Positive Selection Analysis

A phylogenetic species tree was generated using the BirdTree tool [34,35] based on the Ericson all species dataset. Nucleotide protein-guided alignment of avian MDA5 coding sequences was performed using the MAFFT tool [36] with default parameters, and converted back to the nucleotides. Final alignment was manually inspected and edited (File S2). 

Across phylogenetic lineages, positive selection was evaluated using the PAML 4.7 package [37]. A model assuming several groups of residues specified by different *dN*/*dS* for each branch was employed (branch-site model A, model = 2, NSsites = 2) using the codeml program [38]. Specifically, three pairs of hypotheses were compared using Likelihood Ratio Test (LRT): (i) all species in the dataset are under positive selection (alternative) versus no species in the dataset are under positive selection (null); (ii) RIG-I-missing species are under positive selection (alternative) versus no RIG-I-missing species are under positive selection (null); and (iii) RIG-I-missing species are under positive selection (alternative) versus all species in dataset are under positive selection (null). P values corresponding to significance of alternative models were calculated based on LRT statistics. 

Across amino acid sites, FEL, FUBAR and MEME methods [38,39,40], implemented in the HyPhy package, and the BEB [38] method, implemented in PAML 4.7, were used to detect positively selected residues in the MDA5 protein sequence. BEB was performed under the M8 site model. FEL, FUBAR and BEB assume that the selection pressure for each site is constant along the entire phylogeny. On the other hand, MEME is a more specialized method for detecting episodic selection pressure. *p*-values representing the significance of positive selection for each site were calculated using LRT statistics. In the case of BEB and FUBAR, Bayesian posterior probabilities were calculated instead of *p*-values. To avoid false positives, only highly supported PSSs identified based on the consensus of at least three selection methods with probability ≥ 10% were considered in the following analyses. 

PSSs were then compared with previously known MDA5 functional sites and with previously described PSSs in birds and other vertebrates (Data S1). Amino acid substitutions at PSSs were grouped into five physicochemical property groups (acidic, basic, neutral, hydrophobic, and polar) based on Zamyatnin et al. [41] and the key physicochemical properties of these PSSs (molecular charge and hydrophobicity) were determined. We further limited our scope to only physicochemically non-conservative substitutions having potential effect on the protein function. PSSs were plotted on MDA5 domain structure according to Uchikawa et al. [42] and Brisse et al. [12] and visualized by DOG, v. 1.0 [43]. MDA5 sequence logo diagrams were generated using Weblogo 3 application [44].

### 2.3. Structural and Surface Charge Analysis of MDA5

For the chicken 3D model of MDA5 we used a template Protein Data Bank (PDB) ID 5jc7—chicken MDA5 with bound 5′p 24-mer dsRNA and ADP-Mg2+. This model contained amino acids in a range 298–990 (numbering according to GenBank ID: NP_001180567.2). 

To obtain structural information for all MDA5 parts (mainly CARD1 and CARD2 domains), the whole chicken MDA5 structure was also modeled (1–1001 amino acids; numbering according to GenBank ID: NP_001180567.2). For this, homology modeling implemented in Alphafold v2.2 [45] was employed. The best structural model was selected out of five models. The quality of the final model was evaluated using ModFOLD Model Quality Assessment Server v8 [46]. MDA5 3D structures were also modeled for another 18 galliform species (Australian brushturkey, *Alectura lathami*; red-legged partridge, *Alectoris rufa*; Chinese bamboo partridge, *Bambusicola thoracicus*; scaled quail, *Callipepla squamata*; Gunnison grouse, *Centrocercus minimus*; Japanese** quail, *Coturnix japonica*; northern bobwhite, *Colinus virginianus*; brown-eared pheasant, *Crossoptilon mantchuricum; Gallus gallus*; golden pheasant, *Chrysolophus pictus*; rock ptarmigan, *Lagopus muta*; wild turkey, *Meleagris gallopavo*; helmeted guineafowl, *Numida meleagris*; marbled wood quail, *Odontophorus gujanensis*; white-crested guan, *Penelope pileata*; common pheasant, *Phasianus colchicus*; Mikado** pheasant, *Syrmaticus mikado*; greater prairie chicken, *Tympanuchus cupido*; Indian peafowl, *Pavo cristatus*) and 4 anseriform species (swan goose, *Anser cygnoides*; mallard, *Anas platyrhynchos*; tufted duck, *Aythya fuligula*; Muscovy duck, *Cairina moschata*). All models had very good quality in the domain regions (>80; assessed by Alphafold quality score using spectrum b command in PyMol), except for *P. cristatus*. All obtained structural models were superimposed on the chicken MDA5 structure (root mean square deviation, RSMD_mean_ = 0.833 Å).

Identified PSSs were visualized together with previously described functionally relevant sites in PyMol, v2.0.7 (Schrödinger, LLC 2015) on the chicken model. The distance between PSSs and functionally relevant sites was measured using function iterate implemented in PyMol since PSSs in the neighborhood of functionally relevant sites may also co-determine their functional properties. As in Těšický et al. [47], given the putative span of hydrogen bonds, salt bridges [48], and longer-range hydrophobic interactions [49], PSSs were considered to be in close topological proximity to the functional residues only if located < 5Å apart.

Residue solvent exposure (solvent accessibility of a protein residue; RSA) in the chicken model was calculated using the xssp web server (https://www3.cmbi.umcn.nl/xssp/, accessed on 2 June 2022). RSA is defined as the ratio of the residue′s solvent accessible area (ASA) and the corresponding maximum possible solvent accessible area (MaxASA) for a given amino acid [50]. As RSA was highly consistent for the same amino acid between chicken 5jc7 structure and AlphaFold model (Pearson’s r = 0.97, *p* < 0.001), in all our analyses, we only report RSA values from the AlphaFold model. We limited our scope mostly to surface-accessible sites when RSA > 20.0%.

Protein Interaction Property Similarity Analysis (PIPSA; [51]) was employed to determine a matrix of species pairwise surface charge distances in MDA5 structures. Surface electrostatic potential distribution was calculated for all Galloanserae structures in the Adaptive Poisson-Boltzmann Solver (APBS) with a standard environment setup (T = 300 K, ion strength = 50 mM) and visualized using PyMol with the APBS Electrostatics plugin. Cluster analysis of the surface charge was performed in the R software v. 4.1.1 (R Foundation for Statistical Computing, Vienna, Austria) based on the PIPSA distance matrix using pvclust function (package pvclust, clustering method = UPGMA, distance = correlation, nboot = 1000; [52]). Node uncertainty was expressed by Approximately Unbiased bootstrap *p*-values (AU values) and bootstrap probabilities (BP). Electrostatic potential of each structure was visualized in PyMol.

## 3. Results

### 3.1. Multiple Losses of RIG-I and RIPLET in Birds

**Figure 1:**
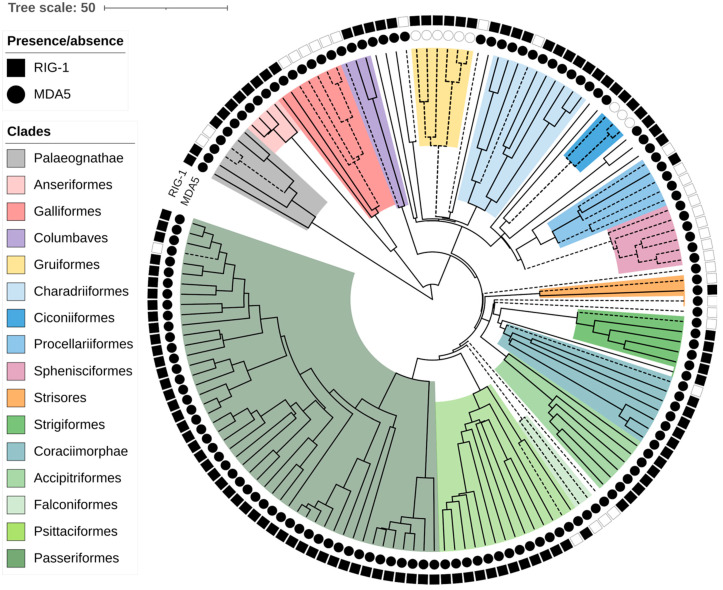
Loss of *RIG-I* and *MDA5* viral sensors in birds. Chronogram of avian evolution is shown for all species analyzed. Each branch tip represents one species. Symbols neighboring the branch tips indicate presence (full) or absence (empty) of the *MDA5* (circles) and *RIG-I* (squares) genes. Dashed lines show lineages where loss of RLR receptor occurs. Avian clades are annotated as described in a legend. Branch lengths scale in millions of years (MYA).

In our previous work, we reported the loss of the *MDA5* virus sensor in two avian lineages [32]. Recently, multiple *RIG-I* losses during avian evolution have been suggested as well [31]. We took advantage of recent progress in the availability and completeness of avian genomic data and collected *RIG-I* and *MDA5* sequences from the genomes of various avian species. When the genes were not annotated in the NCBI database, we attempted to identify the genes’ coding sequences de novo (see details in Section 2). In total, we collected both *RIG-I* and *MDA5* sequences from 148 species representing 28 avian orders (all sequences available in Data S1). We did not identify any additional losses of *MDA5*. In contrast, we observed *RIG-I* absence much more frequently. To make sure that the absence of the genes was not due to the incompleteness of a particular genomic assembly, we classified as *RIG-I* loss only those cases where we observed deleterious mutations in parts of the gene. Such fragments of *RIG-I*, predicted as defective, were identified in 31 species in diverse avian orders (Figure 1, Data S2). To describe the evolutionary aspect of this phenomenon, we used a time-calibrated phylogeny of avian species from the BirdTree project [34,35]. Based on avian phylogeny, the losses presumably represent 16 independent evolutionary events of gene inactivation. Although our sampling was neither uniform nor complete, it is apparent that the gene-loss events occurred over a large evolutionary time span. Based on the minimum age of the common ancestor of *RIG-I*-missing species, the oldest losses occurred 40 million years ago (MYA), for example in Falconiformes. On the other hand, the single detected loss in Passeriformes occurred less than 13 MYA. 

One of the main regulators of RIG-I activity is the RIPLET/RNF135 ubiquitin ligase. Importantly, *RIPLET* had never been identified in the chicken genome [53], which suggested that its presumed absence was associated with the loss of *RIG-I*. In this study, we succeeded in identifying remnants of nonfunctional *RIPLET* in the chicken genome, which confirms its evolutionary loss. Based on this finding, we then assessed the intactness of *RIPLET* CDSs in all avian species missing *RIG-I*. Strikingly, we detected disrupted *RIPLET* genes in all cases of *RIG-I* loss, but almost no disruptions were detected in any other species (Data S1; description of *RIPLET* pseudogenes shown for Galliformes in the next section). The only exceptions were common tern (*Sterna hirundo*) and thick-billed murre (*Uria lomvia*) with apparently intact *RIG-I* and premature stop codons in *RIPLET*. Taken together, these results support the hypothesis on frequent and concerted loss of *RIG-I* together with its regulator *RIPLET* throughout the evolution of birds.

### 3.2. Loss of RIG-I in Galliformes

**Figure 2:**
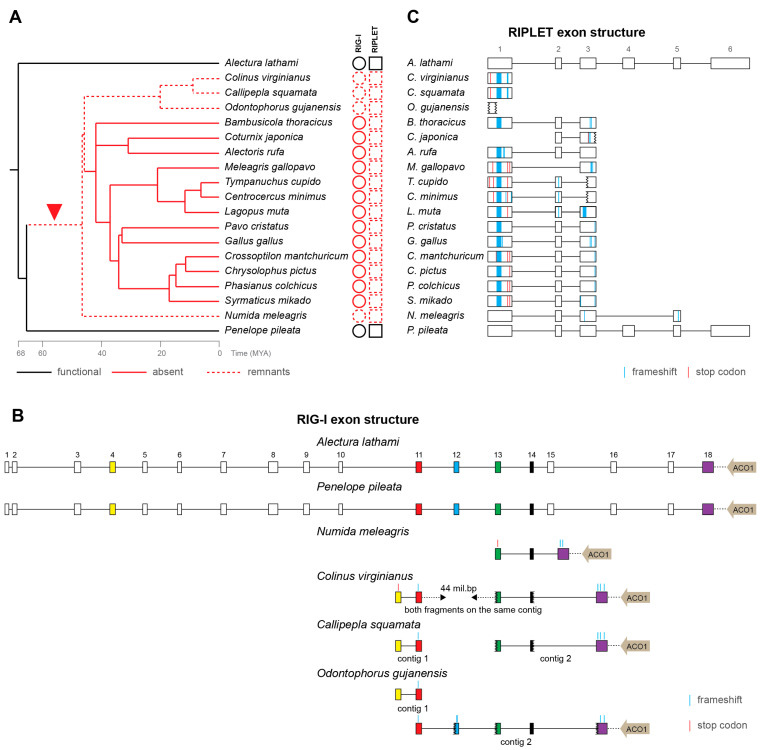
Loss of *RIG-I* and *RIPLET* in Galliformes. (**A**) A time-calibrated phylogenetic tree (from TimeTree.org [61]) of galliform species with the depiction of presence (full black line), remnants (dashed red line) or absence (full red line) of *RIG-I* and *RIPLET* genes, red arrow head indicates the predicted evolutionary time interval of the inactivation event of both genes; MYA—million years ago; (**B**) RIG-I pseudogenization in galliform species. Exons are depicted by boxes, introns by black lines, both to scale. The exons present in the pseudogenes of individual species are marked in color and the predicted inactivating mutations are visualized as described in the legend. The localization of the *ACO1* gene next to the RIG-I sequence is depicted by a gray arrow, and the distance between the two genes is marked by a dashed line, not to scale; (**C**) RIPLET pseudogenization in individual galliform species. Exons are represented by open boxes, introns by black lines, both to scale. The predicted inactivating mutations are visualized as described in the legend.

Loss of *RIG-I* in chicken was already reported more than 10 years ago [29,30], and its functional consequences have been studied intensely [54,55,56,57,58,59]. However, no sequence remnants of chicken *RIG-I* have ever been found. Because some avian genes have been shown to be missing from genomic data for technical reasons—mainly because of high GC content [60]—there has still been the possibility that chicken *RIG-I* might in fact exist. Here, we present strong arguments that *RIG-I* sequences have gradually been lost during the evolutionary history of Galliformes. We show the presence of intact and presumably functional *RIG-I* in two basal galliform species, white-crested guan (*P. pileata*) and Australian brushturkey (*A. lathami*) (Figure 2A). In another four species, helmeted guineafowl (*N. meleagris*), northern bobwhite (*C. virginianus*), scaled quail (*C. squamata*) and marbled wood quail (*O. gujanensis*), we detected highly disrupted *RIG-I* pseudogenes. Indeed, the detected remnants of *RIG-I* orthologs reflect the expected exonic structure and lie in close proximity to the *ACO1* gene, a feature shared by all Galliformes analyzed here (Figure 2A,B) and by mammals. All the remaining galliform species, including chicken, contain no detectable traces of *RIG-I* sequences. Notably, these species, in which *RIG-I* has not been detected at all, form a monophyletic group inside the galliform phylogeny. This pattern is consistent with one initial event of *RIG-I* inactivation after basal galliform speciation and with a consequent gradual loss of its sequence ultimately resulting in its complete absence (or at least undetectability by homology-based searches) in a majority of Galliformes (Figure 2A). Using TimeTree [61], we estimated that the initial inactivation occurred as early as approximately 45–65 MYA.

As mentioned above, the *RIG-I* pseudogenization is almost always coupled with *RIPLET* inactivation in all the avian species we have tested. For Galliformes, we described this in greater detail. All *RIG-I*-defective Galliformes, including the species with no detectable *RIG-I* remnants, contain disrupted *RIPLET* fragments in their genomes (Figure 2C). Besides large deletions, *RIPLET* pseudogenes also possess multiple frameshift and stop-codon mutations. As expected, an apparently functional full-length *RIPLET* sequence was identified in the two basal galliform birds with intact *RIG-I*.

It is important to note that the situation in Galliformes is exceptional; in all other avian clades, we were able to identify at least partial *RIG-I* sequences. Although no traces of pseudogenized *RIG-I* have been found in some galliform birds, the aforementioned lines of evidence strongly support the hypothesis of gene loss during the deep evolution of this clade.

### 3.3. Positive Selection Acting on MDA5 and Its Potential for Compensation of RIG-I Dysfunction

RIG-I and MDA5 recognize structurally similar ligands, which raises the possibility that *MDA5* orthologs functionally compensate for a disrupted *RIG-I* in particular species. To investigate this possibility, we assessed the possible positive selection acting on *MDA5*, which is an indication of its adaptive evolution. Positive selection analysis was performed using a branch-site test of positive selection and its significance was evaluated using a likelihood-ratio test (Table 1). We were able to confirm strong positive selection acting in the whole set of avian *MDA5* orthologs, as well as in the subset of species with nonfunctional *RIG-I* (*p*-value < 0.0001). However, we failed to reveal any increased positive selection in *MDA5* related to the *RIG-I* loss (*p*-value = 1.0000). This suggests no detectable increase of the overall selection pressure that would drive adaptations in *MDA5* following the *RIG-I* loss.

To investigate this in closer detail, we then characterized the individual positively selected sites (PSSs) detected in our dataset of avian *MDA5* sequences. We employed four computational approaches (see Section 2) and obtained 103 PSSs in total (Data S1). To avoid false positives, we considered only highly supported PSSs identified independently by at least three selection methods. This criterion was met in 27 sites, which were then further analyzed. 

**Figure 3:**
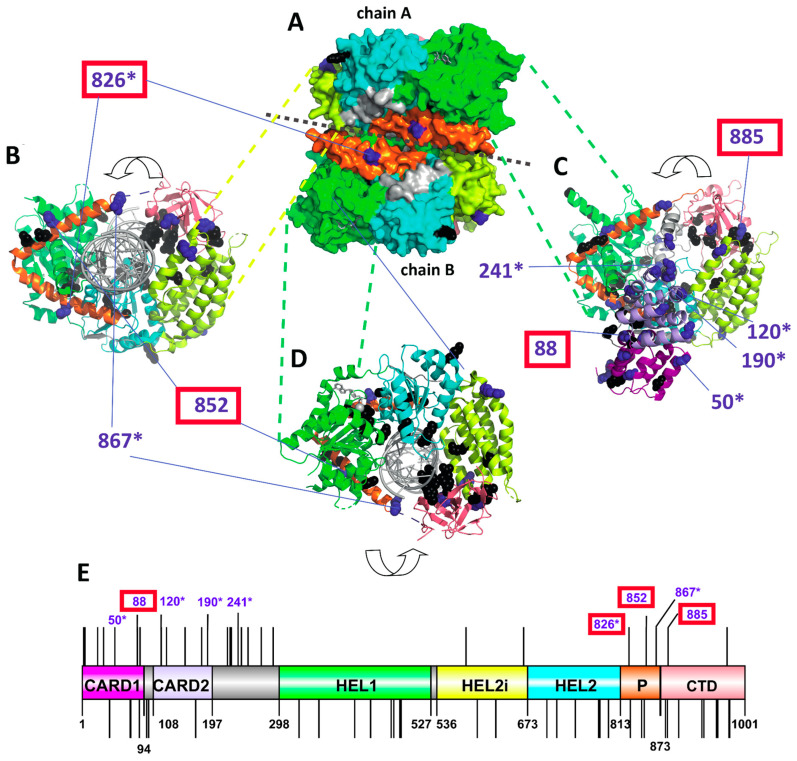
Positively selected sites of avian MDA5 and their position in a protein structure. (**A**–**D**). Three-dimensional structure of chicken MDA5 based on PDB 5jc7 structure with bound 5’p 24-mer double-stranded RNA in grey and ADP-Mg2+: (**A**) side view on a homodimer with shown surface; (**B**) top view on tailed end of chain B; (**C**) Chicken MDA5 structure with CARD1 and CARD2 domains and full CTD domains in its ribbon structure based on Alphaphold modeling (top view on tailed end of chain B); (**D**) bottom view on tailed end of chain B. Previously reported functionally important residues are highlighted in black. PSSs are highlighted in dark blue. Surface-accessible and physicochemically non-conservative PSSs are numbered (numbering is based on the chicken sequence GenBankID: NP_001180567.2). Numbered PSSs located in close proximity to functional sites (<0.5 nm) are highlighted by a red rectangle and sites identified also in other studies are labeled with an asterisk. (**E**) PSSs and functional sites shown on a linear domain structure of chicken MDA5. Upper lines indicate PSS, while the bottom lines indicate functional sites. As in the 3D models, only selected PSS are numbered. CARD1—N-terminal caspase activation and recruitment domain 1 (purple), CARD2—N-terminal caspase activation and recruitment domain 2 (light blue), HEL1—N-terminal RecA-like domain (green), HEL2i—insertion domain (yellow), HEL2—C-terminal RecA-like domain (cyan blue), P—pincer motif (orange), CTD—C-terminal domain (salmon red).

To predict the functional importance of individual PSSs, we mapped them on a 3D model of chicken MDA5, determining their surface availability for ligand binding and their position relative to the predicted functional sites (Figure 3 and Data S1). The majority of PSSs were surface accessible (21 out of 27) and possessed physicochemically non-conservative substitutions (25 out of 27). The largest number of PSSs (12 sites) was found in the two CARD domains, while two were in the helicase domain, three in the pincer domain and two in the CTD of the MDA5 (Figure 3E). Only four PSSs were found close to the residues participating in signaling or binding activity: 88D is close to a site influencing IFN-β and NF-кB promoter-activation; 826N is close to the dsRNA binding site; 852L is close to a residue participating in signaling; and the 885H residue is close to a zinc binding site. Six of the 27 PSSs had also been identified in previous studies as being under positive selection (Data S1 and Figure 3). 

**Figure 4:**
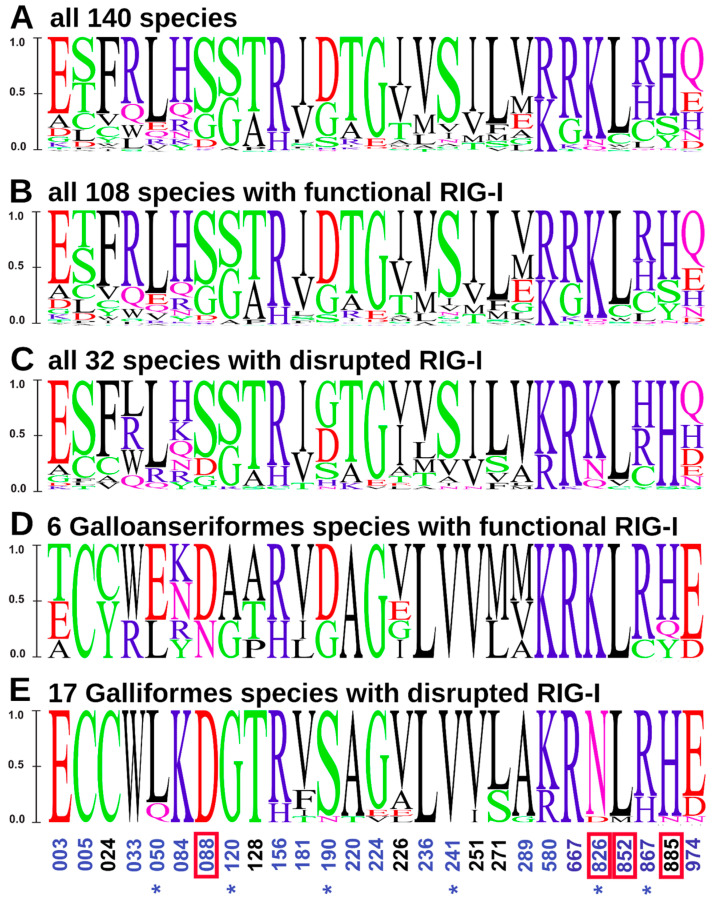
Sequence variation in the positively selected sites of avian MDA5. Letter diagrams show the proportion of specific amino acids in MDA5 (**A**) in all 140 species analyzed; (**B**) in all 108 species with functional RIG-I; (**C**) in all 32 species with disrupted RIG-I; (**D**) in 6 Galloanseriformes species with functional RIG-I; and (**E**) in 17 Galliformes species with disrupted RIG-I. The size of a letter indicates the frequency of a particular amino acid within the sequence alignment. Acidic amino acids are in red, basic in blue, neutral in purple, polar in green and hydrophobic in black. Surface-accessible amino acids assessed from the Alphafold whole-domain model are in blue and PSSs identified also in other studies are labeled with an asterisk. PSSs in proximity to functional sites are highlighted with a red rectangle. Chicken numbering is adopted (GenBank ID NP_001180567.2).

To further inspect amino acid sequence variability at MDA5 PSSs that could be linked to the *RIG-I* loss, we compared the amino-acid substitution diagrams for various subsets of the species analyzed (Figure 4). A comparison between all the species with functional RIG-I and the species without a functional gene showed no marked differences (Figure 4B,C). However, when focusing only on the Galloanserae clade, species with a functional RIG-I differed from those with a non-functional gene in PSS 190 (D/G -> S/N) and PSS 826 (K -> N/D; Figure 4D,E). Interestingly, position 826—where a positively charged lysine is replaced either by an uncharged asparagine or a negatively charged glutamic acid in RIG-I-deficient species—is in close proximity to the dsRNA binding site of the MDA5 protein.

Since the electrostatic potential of a protein can also influence its function and ability to bind ligands, we then compared the surface charge distributions of MDA5 in the RIG-I-functional and RIG-I-deficient species in the Galloanserae lineage. We calculated the surface electrostatic potential distribution (Figure S1) and the species’ pairwise surface charge distances (Figure S2) for all structures. Finally, we performed cluster analysis of the MDA5 surface charge distribution (Figure S3). Although the overall variation of surface charge distribution was relatively low, the two basal galliforms and all anseriforms with functional RIG-I formed a well-supported cluster separated from the cluster of all the remaining galliforms with pseudogenized *RIG-I*.

Taken together, those findings support the idea that some compensatory evolution in MDA5 may occur in species that lost RIG-I.

## 4. Discussion

In this study, we identified multiple independent losses of *RIG-I* during avian evolution. We were inspired by Zheng et al. [31], who made preliminary claims about the absence of RLRs in birds. Here, we analyzed *RIG-I* gene loss in detail using a large number of avian genome assemblies and additional sequencing data. Except for Galliformes, we validated the loss in all the different species by identifying pseudogenized sequences with deleterious mutations. It remains formally possible that in some avian species a second functional *RIG-I* paralog exists; however, we found no indication of such sequences even when analyzing “raw” unassembled sequence reads.

In chicken, conclusive evidence on *RIG-I* loss has been missing because of the absence of an *RIG-I* pseudogene. The possibility remained that *RIG-I* was one of the “hidden genes’’ that are difficult to identify due to technical reasons [60]. Importantly, here we provide additional evidence for the actual absence of *RIG-I* in the chicken genome: (i) an intact *RIG-I* is detectable in two basal galliform species, (ii) gene fragments are present in several more basal lineages of the galliforms (iii) *RIG-I* sequences are undetectable in the remaining galliform species, which form a crown monophyletic clade (iv) *RIPLET* is disrupted in *RIG-I*-lacking galliforms. These findings are consistent with the hypothesis on the initial *RIG-I* and *RIPLET* gene inactivation after basal galliform speciation followed by gradual loss of their sequences. 

There is a striking correlation of *RIG-I* losses with the loss of *RIPLET*; with the two exceptions mentioned above, we have not identified any avian species where only one of these genes is disrupted and the other remains intact. RIPLET is an ubiquitin ligase that activates RIG-I by ubiquitinating its C-terminal region [62], and it appears to be the most important RIG-I regulator [63]. The major reported function of RIPLET is connected to RIG-I (but not to MDA5), although there are some indications of RIG-I-independent functions [62,64,65]. It is, therefore, probable that a *RIPLET* loss is in most cases a consequence of *RIG-I* loss. We can only speculate that the existence of a functional RIPLET in the absence of RIG-I might be redundant or even detrimental. Consistently with this order of gene inactivation, the *RIPLET* orthologs in galliform birds seem to be less disrupted than *RIG-I* in the corresponding species. Of note, all three genes—*RIG-I*, *RIPLET* and *MDA5*—are not genetically linked and reside on different chromosomes. Lastly, *RIG-I* inactivation was reported twice in mammals, namely, in tree shrews and pangolins [24,25]. Our preliminary analysis points to *RIPLET* disruption in these species (data not shown), in line with the correlation seen here in birds. 

We detected positive selection in avian MDA5 in our entire data set, consistently with previous reports of selection forces in avian RLRs [31,55]. We also identified 27 highly confident PSSs, six of which were reported in previous studies (references in Data S1). Four of our PSSs were located in close topological proximity to the annotated functional sites, which could suggest their functional impact. This is in agreement with the general pattern of evolution in other PRR, such as Toll-like receptors (TLRs), showing strong positive selection in birds [27]. The evolutionary loss of a particular gene raises the question of a possible compensation of its function by other gene products. We predict that in those cases the compensating genes should exhibit specific patterns of adaptive evolution linked to the gene-loss events. In our previous study, we did not find any evidence of compensatory evolution of RIG-I in MDA5-deficient avian species. Yet, functional compensation has been suggested in other molecular systems involved in immune defense, including, e.g., the CD1 family in mice [66]. Here, we aimed to document adaptive evolution of MDA5 in RIG-I-deficient species by analyzing potential variation in the strength of positive selection across the avian clades differing in RIG-I presence. While we were not able to show any global pattern differentiating the RIG-I-present and -absent species in strengths of positive selection adaptive, such evolutionary pattern could be masked by the generally pervasive positive selection acting on RLRs. Therefore, our further endeavor focused on variation in individual PSSs in the *MDA5* gene observed in individual RIG-I-lacking clades. In the Galloanserae clade, we observed two PSSs that differed between species with and without a functional *RIG-I* gene. Interestingly, one of them (PSS 826) represents a physicochemically non-conservative substitution in the surface-accessible region. At this site, positively charged lysine is replaced in the RIG-I-lacking species with either an uncharged asparagine or a negatively charged glutamic acid. Since this site is located in close proximity to a dsRNA binding site of the MDA5 protein, this substitution could importantly affect the ligand binding properties of the sensor. Furthermore, our results revealed an unexpected pattern in the MDA5 surface charge distribution within the Galloanserae clade. In contrast to phylogeny, species clustered in the MDA5 surface charge with respect to the presence or absence of functional *RIG-I* gene. These findings support the idea that some compensatory evolution may have occurred in MDA5 after RIG-I loss, probably by targeting a few specific amino acids of MDA5. Yet, until functionally verified, caution is needed since this pattern was observed in a single evolutionary lineage.

Our current work provides a large dataset of *MDA5* sequences from *RIG-I*-positive and negative avian species, together with a set of the PSSs detected. This should make possible further analyses in the future, including in vitro functional tests of MDA5 with introduced substitutions of these residues. One recent analysis identified a PSS (L625E) in the RNA-binding helicase domain of chicken MDA5 [55]. Reciprocal mutagenesis of this site in chicken and human MDA5 showed that this residue determines a more efficient recognition of Newcastle disease virus. One question raised by that work was whether the L625E substitution that causes the acquired function of chicken MDA5 occurred before or after the *RIG-I* loss in the chicken predecessors. Thanks to our identification of basal galliforms with an intact RIG-I, we can now predict that the L625E change occurred before the loss, since it is present in both Australian brushturkey (*A. lathami*) and white-crested guan (*P. pileata*) MDA5.

In general, there can be several reasons for the evolutionary loss of a virus sensor. The relevant pathogen might have disappeared, or it might have developed an effective resistance to the sensor. The loss might have enabled an acquired tolerance to a particular pathogen. Furthermore, the missing sensor could be functionally replaced by a different sensing pathway. The RIG-I losses presented here are intriguing due to their high number, which suggests a long-term continuing tendency for RLR losses during avian evolution. Notably, in humans both MDA5 and RIG-I mutations have been connected to autoimmune disorders caused by the recognition of self RNA structures [67,68]. A tendency to avoid such inappropriate sensing might represent another type of pressure for sensor loss.

In summary, our current work presents a unique evolutionary scenario of multiple independent losses of RLR sensors in birds accompanied by the loss of its regulatory gene, *RIPLET*. We also provide a comprehensive overview of RIG-I loss in the order Galliformes and suggest the possibility of compensatory evolution of MDA5. These findings highlight the dynamic nature of virus RNA sensors and open new avenues for experimental work.

## Figures and Tables

**Table 1:** Significance of positive selection pressure acting on avian MDA5.

Hypothesis	Test of Positive Selection (PAML) ^a^	
	dN/dS (%) ^b^	*p* Value ^c^
Positive selection acting on avian MDA5	2.7 (2.8%)	<0.0001
Positive selection acting on MDA5 of RIG-I-missing avian species	3.5 (1.6%)	<0.0001
Positive selection acting on MDA5 of RIG-I-missing avian species exclusively	-	1.0000

^a^ Branch-site test of positive selection in the codeml program of the PAML package; ^b^ dN/dS ratio estimate of the class of codons under positive selection with the percentage of codons falling into this class designated in parentheses; ^c^ *p* values calculated from likelihood ratio test (LRT) statistics; level of significance is expressed by asterisk.

## Data Availability

Data are contained within the article or Supplementary Material.

## Supplementary Materials

The following supporting information can be downloaded at: https://www.mdpi.com/article/10.3390/v15010003/s1, File S1: Functional coding sequences of RIG-I, MDA5 and RIPLET analyzed in our study. File format is standard FASTA (text file); File S2: MDA5 nucleotide sequence alignment used for analysis of positive selection. File format is standard FASTA (text file); Data S1: List of positively selected sites in MDA5 avian orthologs and their properties. File format is Microsoft Excel XLSX; Data S2: Surface electrostatic potential distribution distance similarity matrix of MDA5 in Galliformes and Anseriformes based on webPIPSA analysis. File format is Microsoft Excel XLSX; Figure S1: Detailed phylogram of species analyzed in our study with indication of RIG-I and MDA5 presence/absence. Phylogram is obtained from BirdTree.org. Full or empty circles/squares next to branch tips represent presence or absence of MDA5/RIG-I gene. Time scale in million years ago (MYA) is shown. File format is PNG; Figure S2: Surface electrostatic potential distribution of MDA5 structure in Anseriformes and Galliformes. File format is PDF; Figure S3: The hierarchical cluster analysis of surface electrostatic potential of MDA5 structure in Anseriformes and Galliformes. File format is PDF.
